# Appraisal of the daily clinical rounds performed in an open and general ICU

**DOI:** 10.1186/cc14670

**Published:** 2015-09-28

**Authors:** Roberto R Filho, Ana Maria Cavalheiro, Denise C Cazati, Frederico Lomar, Gustavo FJ de Matos, Murillo SC de Assunção, Roberto R Filho, Tatiana Mohovic, Thiago D Correa

**Affiliations:** 1 Department of Intensive Care Medicine, Hospital Israelita Albert Einstein, Morumbi, São Paulo, São Paulo, Brazil

## Introduction

Daily multidisciplinary clinical rounds involving physicians, nurses, respiratory therapists, nutritionists and clinical pharmacists improve the quality and outcomes of ICUs [1]. However, data regarding performance of these clinical rounds in an open-ICU model are limited.

## Objective

To address the characteristics and the main interventions proposed and made during multiprofessional clinical rounds performed in a clinical-surgical open ICU.

## Methods

This observational study was conducted in a 41-bed open clinical-surgical ICU of a tertiary-care, private hospital in São Paulo, Brazil. From February 20 through March 28 2013, demographic data, SAPS 3, the participants of the ICU clinical rounds, the number and type of the proposed interventions, and the number of performed interventions by the multidisciplinary team were recorded and analyzed.

## Results

A total of 158 clinical rounds were included in this analysis. Fifty-four percent (85/158) of the patients were male with median (IQR) age of 73 (60-84) years and SAPS 3 score of 52 (44-65). The multidisciplinary team was composed of a senior physician (157/158 (99%)), nurses (157/158 (99%)), an on-call staff physician (150/158 (95%)), respiratory therapists (149/158 (94%)), a clinical pharmacist (89/158 (56%)) and nutritionists (62/158 (39.2%)). The median (IQR) number of interventions proposed during the multidisciplinary rounds was 1 (0-2) and the number of performed interventions was 1 (0-2) (Table 1). Interventions were more frequently proposed by senior physicians (82/158 (52%)) followed by respiratory physiotherapists (43/158 (27%)) and a clinical pharmacist (29/158 (18%)).

## Conclusion

In our open ICU model where decisions should be shared with assistant doctors, the implementation of daily clinical rounds was associated with an intense participation of the multidisciplinary team and with a high level of performance of the proposed interventions. These actions are probably associated with better care of the critically ill patients. However, further studies are needed to correlate such interventions with clinical outcomes.

**Table 1:** Main interventions proposed and performed during the multiprofessional rounds.

Main intervention	Proposed	Performed
Changes in nutritional support	39/158 (24.7%)	39/39 (100.0%)
Early mobilization	37/158 (23.4%)	35/37 (94.6%)
Adjustments on sedation or analgesia	51/158 (32.3%)	49/51 (96.1%)
Adjustments on	26/158 (15.8%)	26/26 (100.0%)
Withdrawal of invasive devices	23/158 (14.6%)	19/23 (82.6%)
Adjustments of ventilatory parameters	23/158 (14.6%)	23/23 (100.0%)
Adjustments on glycemic control	25/158 (14.6%)	25/25 (100.0%)
Deep vein thrombosis prophylaxis	13/158 (8.2%)	12/13 (92.3%)
Stress ulcer prophylaxis	6/158 (3.8%)	5/6 (83.3%)

Values represent *n *(%).

## References

[B1] Kim MM Barnato AE Angus DC Fleisher LA Kahn JM The effect of multidisciplinary care teams on intensive care unit mortality Arch Intern Med 2010 170 4 369 376 20177041 10.1001/archinternmed.2009.521 PMC4151479

[B2] Weiss CH Moazed F McEvoy CA Singer BD Szleifer I Amaral LA Prompting physicians to address a daily checklist and process of care and clinical outcomes: a single-site study Am J Respir Crit Care Med 2011 184 6 680 686 10.1164/rccm.201101-0037OC 21616996 PMC3208596

